# Core and bridge symptoms of demoralization in Chinese female cancer patients: a network analysis

**DOI:** 10.3389/fpsyt.2024.1273411

**Published:** 2024-02-05

**Authors:** Yijing Gong, Bin Shang, Jianing Tan, Caifeng Luo, Zekun Bian, Xiaoxiang Wu, Tingting Fan, Qian Zhao, Lili Liu, Weiyi Sun

**Affiliations:** ^1^ School of Medicine, Jiangsu University, Zhenjiang, China; ^2^ Department of Neurology, Changshu Second People’s Hospital, Suzhou, China; ^3^ Department of Breast Surgery, Nanjing Maternity and Child Care, Nanjing, China; ^4^ Department of Oncology, Zhenjiang First People’s Hospital, Zhenjiang, China; ^5^ Department of Thyroid and Breast Surgery, Affiliated Hospital of Jiangsu University, Zhenjiang, China; ^6^ Department of Obstetrics and Gynecology, Nanjing Drum Tower Hospital, Nanjing, China

**Keywords:** female, cancer patient, demoralization, network analysis, core symptom, bridge symptom

## Abstract

**Objective:**

In this study, we explore the core and bridge symptoms of demoralization in female cancer patients in China, and provide a basis for precise psychological intervention among female cancer patients.

**Methods:**

This study used a cross-sectional survey. Participants were recruited from three third-class hospitals in Jiangsu Province from June 2022 to June 2023 using the convenience sampling method. The severity of each symptom of demoralization was investigated in female cancer patients using the Demoralization Scale (DS). Network analysis was performed using the R language to identify core and bridge symptoms in the network and further explore some characteristic edge connections in the network.

**Results:**

The network structure model of demoralization had strong accuracy and stability. In the network, the symptoms with the highest strength centrality were “Discouragement” (C3, strength=2.19), “No self-worth” (A3, strength=1.21), “Don’t want to live” (A5, strength=1.20), “Hopeless” (D4, strength=0.81), and “Vulnerability” (B3, strength=0.74), respectively. The bridge strength analysis identified “Hopeless” (D4, bridge strength=0.92), “Discouragement” (C3, bridge strength=0.85), “No self-worth” (A3, bridge strength=0.75), “Poor spirits” (E2, bridge strength=0.71), and “Vulnerability” (B3, bridge strength=0.69) as the bridge symptoms. The strongest edge connections of all dimensions were “No self-worth” and “Worthless” (A3—E6, edge weighting=0.27), “Poor spirits” and “Loss of emotional control” (E2—D1, edge weighting=0.22), “Discouragement” and “Vulnerability” (C3—B3, edge weighting=0.14), and “Hopeless” and “No meaning of survival” (D4—A4, edge weighting=0.12).

**Conclusion:**

“Discouragement (C3)”, “No self-worth (A3)”, “Hopeless (D4)”, and “Vulnerability (B3)” are both core symptoms and bridge symptoms. These symptoms can not only trigger a patient’s demoralization but also stimulate more severe symptom clusters through interactions. The early recognition of and intervention regarding these symptoms could be important for the prevention and treatment of demoralization among female cancer patients.

## 1 Introduction

According to the World Health Organization’s International Agency for Research on Cancer (IARC) report, in 2020, new cancer cases and death rates in China ranked first in the world, and the age-standardized incidence of cancer in women was on the rise (1, 2). Survival rates for women with cancer are statistically about 50% higher than those for men (1). This longer survival period with cancer makes the psychological vulnerability of female cancer patients more pronounced as they face cancer treatment and carry the disease for longer. Oona et al. showed that the severity of psychological problems in female cancer patients was significantly higher than that in males (3). During cancer diagnosis, treatment, and recovery, psychological distress can occur on a continuum from normal fear, worry, or sadness to severe anxiety, depression, and existential despair (4). With the transformation of the traditional medical model into a biopsychosocial medical model, the psychological problems of cancer patients have received increasingly more attention from the medical community (5). Therefore, how to help female cancer patients maintain a healthy psychological state deserves to be explored more thoroughly. Previous studies have focused on the current status and influencing factors of personal control and mental disorders in female cancer patients, but few studies have focused on the important impact of demoralization on the psychological problems of female cancer patients.

Both demoralization and depression are common in cancer and are characterized by disturbed sleep and appetite and suicidal ideation. Depression is featured by anhedonia. Depression patients strive to experience expected and perfect joy while demoralization patients can still experience perfect joy (6). Demoralization is a mental state in which the patient has a persistent inability to cope along with feelings of helplessness, despair, meaninglessness, subjective incompetence, and decreased self-esteem against a background of internally or externally induced stress (7). Demoralization is a common psychological problem among cancer patients (8), with a prevalence of up to 13%-18% (8). Oona et al. showed that the degree of demoralization in female cancer patients was significantly higher than that in males (3); thus, early identification and effective intervention are of great clinical significance for female cancer patients. Yu et al.’s study systematically identified, evaluated, and synthesized the relationship between demoralization and related factors in cancer patients based on bio-psychosocial models (9) but lacked a detailed analysis of the specific effects of subtle symptoms within demoralization. Vehling et al. (10) explored the co-occurrence and independence between the demoralization and other psychiatric disorders and described the impact of demoralization on the single symptom of suicidal ideation; however, the study lacked research on the importance of each symptom of demoralization and their interactions. In addition, previous studies have used structural equations, latent category analysis, or total scores to determine the severity of demoralization. It’s a pity that both the traditional latent categorical and latent dimensional models of mental disorder view symptoms as responses to the underlying disease entity rather than factors contributing to mental disorders (11). Therefore, the latent variable can be seen as a mediator that transfers effects from stressors to symptoms. Accordingly, in latent variable models, external variables are typically modeled as conditionally independent symptoms, given the latent variable (12). There are other methods that ignore the causal relationship between observed indicators, such as the reflective model relies on the assumption that there is no direct causal relationship between variables, however, causal relations between observables are likely to exist in many psychological constructs (13).These methods do not allow one to observe subtle correlations between specific psychological symptoms (e.g., meaninglessness, worthlessness, and discouragement).In other words, it is difficult to adopt precise psychological treatments for different symptom subtypes of demoralization using traditional methods.

In recent years, network analysis has been increasingly used in the fields of psychology and psychiatry (14). In this method, each network consists of nodes and edges. Nodes represent symptoms, domains, or constructs, while edges represent partial correlations between these nodes (15). The thickness and saturation of the edges correspond to the strength of the partial correlation: The stronger the correlation is, the thicker and more saturated the edges become (16). Network analysis also provides indicators for centrality, indicating the importance of any symptom in the network. These indicators can be used to identify core symptoms in the network (17). Core symptoms have the strongest correlation with other symptoms. By accurately identifying core symptoms, determining the interconnections between core symptoms and other symptoms, and targeting the development of effective interventions, we can more accurately prevent the occurrence of serious consequences. Yang et al.’s study applied network analysis to explore the network correlates for fear of cancer recurrence, anxiety, and depression in breast cancer patients (14). However, the authors’ approach of constructing networks for multiple psychological variables may have diminished the advantages of network analysis in allowing precise measurements. Another German study investigated the interrelationship and relative importance of demoralization symptoms in patients using network analysis (18), but this study included all cancer groups and did not examine the specific population of women, making the results lack specificity. In addition, the study population included only German cancer patients. Thus, due to differences between cancer types and cultures, the results may not be applicable to Chinese female cancer patients.

In summary, to understand the interactions and relative importance of the symptoms of demoralization in Chinese female cancer patients, we need more in-depth investigations to overcome the limitations of previous studies. The present study attempts to explore the core symptoms, bridge symptoms between different psychological subgroups, and interactions between the symptoms of demoralization in Chinese female cancer patients using the approach of a network structure model. To our knowledge, this is the first study to use network analysis to explore demoralization among Chinese female cancer patients.

## 2 Materials and methods

### 2.1 Study design and participants

The study was conducted from June 2022 to June 2023 using the convenience sampling method to select 413 eligible female cancer patients in three tertiary hospitals in Nanjing and Zhenjiang, Jiangsu Province. For this study, we conducted a multi-center, cross-sectional survey. Face-to-face questionnaires were used for data collection. The inclusion criteria were as follows: 1) female; 2) adult (18 years of age or older); 3) diagnosed with breast cancer and aware of the diagnosis; 4) native speaker of Chinese with literacy; and 5) conscious enough to provide written informed consent. The exclusion criteria were as follows: 1) the presence of major diseases other than cancer; and 2) cognitive dysfunctions or intellectual disabilities that could affect the validity of this study. The protocol was approved by the Medical Ethics Committee of Jiangsu University under approval number NO.20220511-1.

Before the investigation, the researchers were trained in a standardized manner using unified guidance language to explain the purpose and precautions of the investigation in detail to the research subjects. The research subjects were also required to sign informed consent to participate in the survey. The survey followed the principle of voluntary anonymity. In addition, the patient could withdraw at any time if he or she wished to do so. After filling out the questionnaire, the responses were collected on the spot. Then, the researcher checked whether the questionnaire was completed and, if not, asked the patient to finish the remainder before collecting the results. After the survey, the researchers checked the quality of the questionnaire responses and excluded obviously invalid questionnaires such as the exact same answers given before and after. The qualified questionnaires were sorted and entered, and then the entry results were checked by another researcher to avoid entry errors.

### 2.2 Measurements

#### 2.2.1 Demographic information and disease characteristics

The researchers included sociodemographic and disease-related data according to the purpose of the study combined with relevant literature. Demographic data included occupational status, age, marital status, education level, financial burden, primary caregiver, payment method, and place of residence. Disease-related information included tumor type, length of diagnosis, and current stage of treatment.

#### 2.2.2 Demoralization scale

The scale was developed by Kissane et al. (19), with a Cronbach’s α coefficient of 0.946. In this study, the revised Demoralization Scale Mandarin Version (DS-MV) by Liu Peipei et al. (20) was adopted, and the Cronbach’s α coefficient was 0.902. This scale consists of five dimensions: disheartenment, loss of meaning, helplessness, sense of failure, and dysphoria. The sense of failure dimension contained 6 items, the disheartenment and loss of meaning dimensions each contained 5 items, and the dysphoria and helplessness dimensions each contained 4 items, for a total of 24 items. A 5-point Likert scale ranging from “strongly disagree” to “strongly agree” was used to score responses on a scale from 0 to 4. The total scores ranged from 0 to 96, with higher scores indicating higher levels of patient demoralization. The scale developers suggested a score of 30 as the threshold, and those with scores greater than 30 were considered to have a significant level of demoralization.

### 2.3 Statistical analysis

We used SPSS version 26.0 for descriptive statistics and different R packages in R version 4.2.0 for network analysis. Three main analyses were performed: network estimation, centrality and predictability measures, and accuracy and stability estimations. All network visualizations are presented using the Fruchterman–Reingold algorithm from the qgraph package.

#### 2.3.1 Network estimation

For estimating and visualizing the network, we used the “qgraph” (version 1.9.2) and “bootnet” (version 1.5.0) software in the R package to perform the analysis (15). The network structure was estimated using the graph least absolute shrinkage and selection operator (gLASSO) method (21), based on the Extended Bayesian Information Criterion (EBIC), resulting in a sparse and simple network model that was more interpretable than the original model (22). Psychological network analysis was used to express the centrality and interaction relationship of various psychological symptoms through the network structure model. The proposed network structure model features a collection of different symptom groups composed of two parts, where the “node” represents the observed psychological symptoms, and “edge” represents the interaction between the observed psychological symptoms. The thickness of each edge represents the degree to which the nodes influence each other; the greater the correlation between the two nodes, the thicker the edge. The colors of the edges indicate the direction of the correlation. Positive correlations are connected with blue edges, while negative correlations are connected with red edges. Nodes that exhibit stronger correlations with other nodes are located more centrally in the network (23).

#### 2.3.2 Centrality and predictability measurements

In network analysis, centrality is typically evaluated using three metrics: strength, betweenness, and closeness (15). A higher index value means that the node is more important in the network (15). Previous studies have found that closeness and betweenness are not sufficiently stable to measure the centrality of nodes (24). Thus, in this study, we emphasized the network centrality measure of node strength (25), which is the sum of the absolute values of the correlations between one node and the other nodes in the network structure model (14). Strength centrality indicates the likelihood that the activation of a particular node will subsequently activate other nodes (26). The higher the strength of the node, the stronger the centrality of the corresponding symptom and the more important its impact on other symptoms. The predictability of each node was estimated using the R package “mgm” (versions 1.2-12) (21). Predictability is defined as the variance in a node explained by all other nodes in the network (27). Bridge strength centrality was used to identify bridge symptoms, which are broadly defined as symptoms that connect different symptom clusters (28). This study used the R package networktools (version 1.5.0) to identify bridge symptoms and calculate the bridge strength centrality index connecting the five symptom clusters. The strong causal relationship between the bridge symptoms and other symptom clusters reflected the extent to which the current symptoms were connected to the other clusters and influenced the development of other symptom clusters. There was a strong causal relationship found between bridge symptom and other symptom clusters, reflecting the close connection between current symptoms and other cluster symptoms and indicating a significant influence on the development of other symptom clusters.

#### 2.3.3 Estimating the accuracy and stability of the network

Based on the proposal of Epskamp et al. (15), the robustness of the network solution was evaluated using the R package “bootnet” (version 1.5.0) to estimate the accuracy of edge weights and the stability of the centrality index (21). We estimated the accuracy of network edges based on bootstrapped 95% confidence intervals (CIs) of the edge weights. The smaller the overlap between 95% confidence intervals, the more accurate the edge estimate (16). Then, the case-dropping subset bootstrap was executed to calculate the centrality stability coefficient (CS-C) and thereby assess the stability of the centrality index (26). The CS-C value represents the maximum proportion of samples that can be excluded while ensuring, with 95% probability, that the correlation between the original centrality indices will be at least 0.70 (15). In general, the CS-C should be no less than 0.25 and ideally above 0.50 (21).

## 3 Results

### 3.1 Participants’ general characteristics, disease-related information, centrality indexes, predictability indexes, and symptom severity

**Table 1** shows the participants’ characteristics. A total of 420 female cancer patients met the study criteria and participated in completing the questionnaires. In total, 418 questionnaires were recovered, 5 invalid questionnaires were excluded, and 413 valid questionnaires were ultimately included, with an effective recovery rate of 98.33%. The mean age of the survey subjects was 54.01 ± 10.35 years (range: 26-84 years). The majority of the participants were married (n=398, 96.4%) and had an education beyond elementary school (n=336, 81.4%). There were 106 in-service staff (25.7%), and 307 unemployed or retired staff (74.3%) represented in the sample. Most patients lived in cities (n=264, 63.9%), and most caregivers were spouses (n=245, 59.3%). The vast majority of the respondents were breast cancer patients (n=261, 63.2%), and most of the patients were in the treatment stage of chemoradiotherapy (n=327, 79.2%). (See **Table 1** for details).

**Table 1:** General characteristics of female cancer patients (n=413).

Variables	n(%) or Mean ± SD
Occupation	
Unemployed	197 (47.7)
Be employed	106 (25.7)
Retired	110 (26.6)
Age	54.01 ± 10.35
Marital status	
Unmarried	2 (0.5)
Married	398 (96.4)
Divorce	5 (1.2)
Widowed	8 (1.9)
Education level	
Primary school and below	77 (18.6)
Junior high school	202 (48.9)
High school or technical secondary school	82 (19.9)
Junior college	33 (8.0)
Bachelor degree or above	19 (4.6)
Economic pressure	
Higher	78 (18.9)
General	148 (35.8)
Lower	187 (45.3)
Primary caregiver during illness	
Spouse	245 (59.3)
Child	73 (17.7)
Parents	14 (3.4)
Other	41 (9.9)
None	40 (9.7)
Methods of payment of medical expenses	
Own expense	2 (0.5)
Farmer’s insurance	162 (39.2)
Employee medical insurance	151 (36.6)
Medical insurance for urban and rural residents	98 (23.7)
Residence	
Countryside	126 (30.5)
City	264 (63.9)
Town	23 (5.6)
Length of diagnosis(month)	15.19 ± 25.65
Current stage of treatment	
Surgery	43 (10.4)
Chemoradiotherapy	327 (79.2)
Other	43 (10.4)
Cancer type	
Breast cancer	261 (63.2)
Gynecological Cancer	76 (18.4)
Cancer of the digestive system	44 (10.7)
Other	32 (7.7)

**Table 2** shows the severity of all symptoms. The results showed that “Irritable (B2)” (2.42 ± 1.24) was the most severe symptom in the demoralization network structure model, “Fall into difficulty (C5)” (2.09 ± 1.38) was the second most severe psychological symptom, and “Guilt (B1)” (2.09 ± 1.41) ranked third. “Sadness (C2)” (2.08 ± 1.31) and “Regret (E4)” (1.98 ± 1.36) ranked fourth and fifth, respectively. The symptoms that ranked first and third, belonged to the “Dysphoria” dimension, while the second and fourth symptoms belonged to the “Disheartenment” dimension.

**Table 2:** Centrality, bridge centrality, predictability, severity indices and abbreviations for each symptom in demoralization.

symptoms	abbreviations	mean	standard deviation	prevalence	Strength (Z-scores)	predictability	bridge strength
1. I can’t do many things of value for others.	E1 No social value	1.15	0.88	11.4	0.34	0.45	0.62
2. My life seems to be pointless.	A1 Meaningless	0.75	0.76	4.4	0.59	0.56	0.50
3. My life has no purpose.	A2 No target	1.61	1.34	36.6	-1.60	0.27	0.54
4. My role in life has been lost.	A3 No self-worth	0.77	0.87	6.8	1.21	0.51	0.75
5. I no longer feel emotionally in control.	D1 Loss of emotional control	1.08	0.99	12.1	-0.52	0.45	0.55
6. I am not in good spirits.	E2 Poor spirits	1.00	0.95	12.1	0.71	0.56	0.71
7. No one can help me.	D2 No effective assistance	1.03	1.06	13.6	-0.71	0.34	0.41
8. I feel that I cannot help myself.	D3 Self-help difficult	0.94	0.98	12.3	-0.04	0.44	0.55
9. I feel hopeless.	D4 Hopeless	1.15	1.13	20.3	0.81	0.49	0.92
10. I feel guilty.	B1 Guilt	2.09	1.41	60.5	-0.95	0.42	0.49
11. I feel irritable.	B2 Irritable	2.42	1.24	68.8	-2.02	0.21	0.35
12. I can’t cope fairly well with life.	E3 Cope difficult	1.10	1.10	17.9	0.43	0.46	0.70
13. I have a lot of regret about my life.	E4 Regret	1.98	1.36	51.6	-0.97	0.31	0.55
14. Life is no longer worth living.	A4 No meaning of survival	0.60	0.72	2.9	0.56	0.59	0.26
15. I tend to feel hurt easily.	B3 Vulnerability	1.79	1.37	49.6	0.74	0.49	0.69
16. I am angry about a lot of things.	B4 Wrath	1.36	1.21	26.6	0.64	0.44	0.55
17. I’m not proud of my accomplishments.	E5 No accomplishment	1.59	1.43	34.4	-1.25	0.26	0.29
18. I feel distressed about what is happening to me.	C1Distress	1.94	1.34	46.2	-0.86	0.36	0.37
19. I’m not a worthwhile person.	E6 Worthless	1.20	1.10	14.0	-0.37	0.34	0.46
20. I would rather not be alive.	A5 Don’t want to live	0.58	0.63	1.5	1.20	0.67	0.37
21. I feel sad and miserable.	C2 Sadness	2.08	1.31	51.1	0.28	0.39	0.39
22. I feel discouraged about life.	C3 Discouragement	1.62	1.35	44.3	2.19	0.68	0.85
23. I feel quite isolated or alone.	C4 Loneliness	1.58	1.38	41.9	0.23	0.56	0.44
24. I feel trapped by what is happening to me.	C5 Fall into difficulty	2.09	1.38	56.7	-0.64	0.43	0.31

### 3.2 Network analysis

#### 3.2.1 Network structure

**Figure 1:**
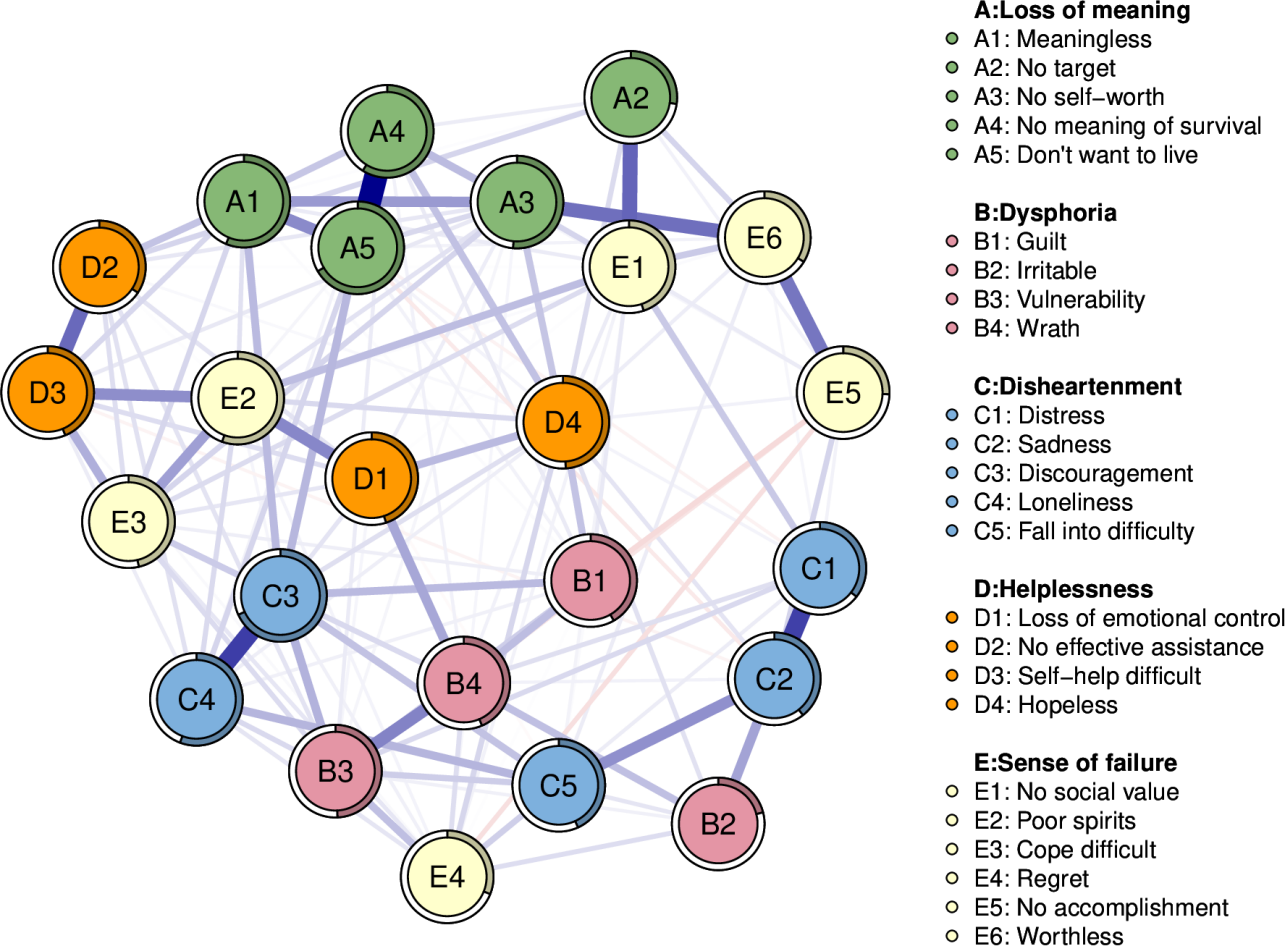
Network structure model of demoralization in female cancer patients. The five different colors represent five different clusters of psychological symptoms that cause Demoralization. The blue edges represent positive correlations and red represent negative correlations. Edge thickness represents the strength of the connection between symptoms.

**Figure 1** visualizes the network structural model showing the interactions of symptom clusters for the five dimensions of demoralization. The network structure model measured a total of 276 edges, including 136 non-zero edges, which accounted for 49.3% of all edges. The model results show that the nodes in the symptom clusters of the five dimensions were positively correlated in their respective network clusters, except for the dimension of “Sense of failure”, whereas both positive and negative correlations were observed between the nodes in the different dimensions. The model and edge weights in each dimensions show that connecting “No meaning of survival” and “Don’t want to live” (A4—A5, edge=0.48), “Discouragement” and “Loneliness” (C3—C4, edge=0.36), “No effective assistance” and “Self-help difficult” (D2—D3, edge=0.28), “No accomplishment” and “Worthless” (E5—E6, edge=0.26), “Vulnerability” and “Wrath” (B3—B4, edge=0.23) had the strongest positive edges.

#### 3.2.2 Centrality and predictability

**Figure 2:**
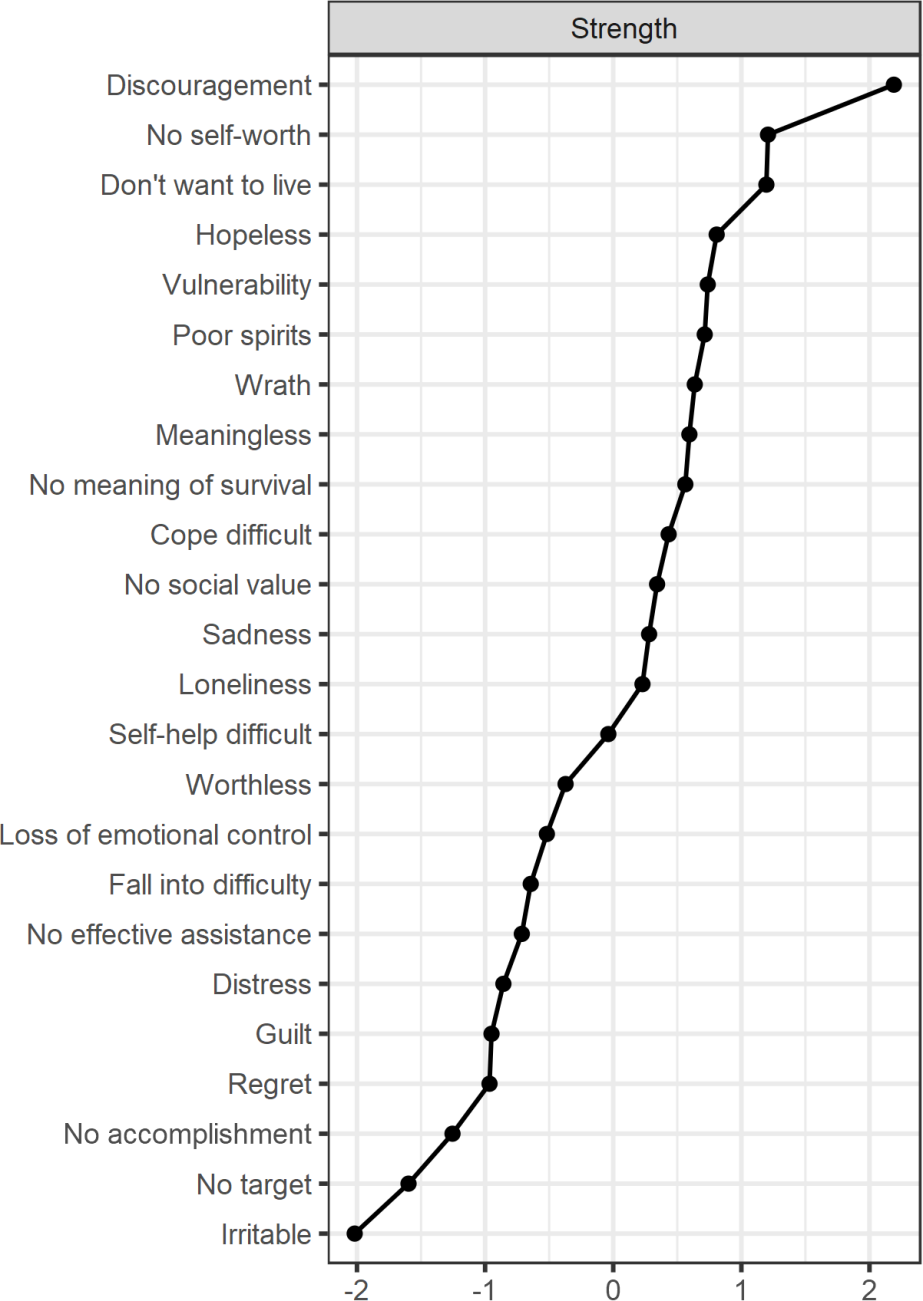
The centrality index of each node in the network structure model. Strength measures the importance of the nodes. (Z-scores).

**Figure 2** shows the centrality index of the network. The symptom “Discouragement” (C3, strength=2.19) was the most central symptom in the whole network, followed by the symptom of “No self-worth” (A3, strength = 1.21). The third, fourth, and fifth most central symptoms were “Don’t want to live” (A5, strength = 1.20), “Hopeless” (D4, strength=0.81), and “Vulnerability” (B3, strength=0.74). As shown in **Table 2**, the five most predictable nodes in this network were, in order, “Discouragement” (C3, predictability=0.68), “Don’t want to live” (A5, predictability=0.67), “No meaning of survival” (A4, predictability=0.59), “Meaningless” (A1, predictability=0.56), and “Loneliness” (C4, predictability=0.56).

#### 3.2.3 Bridge centrality and bridge connections

**Figure 3:**
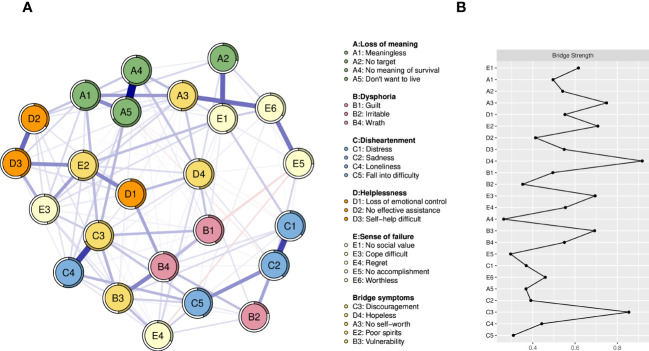
**(A)** Network structure model containing bridge symptoms, the yellow nodes represent bridge symptoms. **(B)** Centrality index of bridge symptoms. (Z-scores).

As shown in **Figure 3**, the symptoms “Hopeless” (D4, bridge strength=0.92), “Discouragement” (C3, bridge strength=0.85), “No self-worth” (A3, bridge strength=0.75), “Poor spirits” (E2, bridge strength=0.71), and “Vulnerability” (B3, bridge strength=0.69) are bridge symptoms connecting the five symptom clusters. The specific values of the edge weights show how closely the dimensions of the bridge symptoms are connected. According to the measurements of the edge weights, the strongest bridge connections were “No self-worth” and “Worthless” (A3—E6, edge weight=0.27), followed by “Poor spirits” and “Loss of emotional control” (E2—D1, edge weight=0.22), then”Discouragement” and “Vulnerability” (C3—B3, edge weight=0.14), finally “Hopeless” and “No meaning of survival” (D4—A4, edge weight=0.12). Moreover, the edge weights for all symptoms in the network are shown in **Table 3**.

**Table 3:** All estimated edge weights.

	B1	B2	B3	B4	B5	B6	B7	B8	B9	B10	B11	B12	B13	B14	B15	B16	B17	B18	B19	B20	B21	B22	B23	B24
B1	0.00	0.04	0.29	0.08	0.00	0.12	0.00	0.00	0.04	0.04	0.00	0.07	0.03	0.00	0.00	0.00	0.04	0.10	0.10	0.00	0.00	0.00	0.00	0.02
B2	0.04	0.00	0.01	0.19	0.00	0.06	0.11	0.08	0.00	0.01	0.00	0.08	0.00	0.11	0.00	0.00	0.00	0.00	0.00	0.22	0.00	0.13	0.00	0.00
B3	0.29	0.01	0.00	0.00	0.00	0.00	0.08	0.00	0.07	0.00	0.00	0.00	0.00	0.04	0.00	0.00	0.03	0.00	0.08	0.00	0.00	0.00	0.00	0.00
B4	0.08	0.19	0.00	0.00	0.03	0.05	0.04	0.04	0.11	0.00	0.00	0.08	0.02	0.13	0.02	0.00	0.00	0.00	0.27	0.07	0.00	0.00	0.00	0.00
B5	0.00	0.00	0.00	0.03	0.00	0.22	0.05	0.06	0.13	0.00	0.00	0.05	0.00	0.00	0.02	0.17	0.00	0.00	0.00	0.00	0.00	0.05	0.00	-0.01
B6	0.12	0.06	0.00	0.05	0.22	0.00	0.00	0.21	0.07	0.00	0.00	0.18	0.02	0.00	0.00	0.00	0.00	0.00	0.01	0.00	0.00	0.02	0.07	0.00
B7	0.00	0.11	0.08	0.04	0.05	0.00	0.00	0.28	0.01	0.00	0.00	0.06	0.00	0.00	0.05	0.00	0.00	0.00	0.00	0.00	0.00	0.00	0.06	0.00
B8	0.00	0.08	0.00	0.04	0.06	0.21	0.28	0.00	0.00	0.00	0.00	0.15	0.01	0.00	0.00	0.00	0.00	0.00	0.00	0.00	-0.02	0.00	0.04	0.00
B9	0.04	0.00	0.07	0.11	0.13	0.07	0.01	0.00	0.00	0.11	0.06	0.00	0.08	0.12	0.00	0.03	0.03	0.02	0.03	0.02	0.05	0.06	0.03	0.00
B10	0.04	0.01	0.00	0.00	0.00	0.00	0.00	0.00	0.11	0.00	0.00	0.03	0.00	0.04	0.09	0.12	-0.07	0.00	0.00	0.02	0.00	0.13	0.03	0.01
B11	0.00	0.00	0.00	0.00	0.00	0.00	0.00	0.00	0.06	0.00	0.00	0.00	0.06	0.00	0.03	0.12	0.00	0.01	0.00	0.00	0.17	0.00	0.00	0.05
B12	0.07	0.08	0.00	0.08	0.05	0.18	0.06	0.15	0.00	0.03	0.00	0.00	0.05	0.00	0.07	0.07	0.00	0.00	0.00	0.02	0.00	0.10	0.00	0.00
B13	0.03	0.00	0.00	0.02	0.00	0.02	0.00	0.01	0.08	0.00	0.06	0.05	0.00	0.00	0.12	0.06	-0.06	0.04	0.00	0.00	0.00	0.03	0.05	0.09
B14	0.00	0.11	0.04	0.13	0.00	0.00	0.00	0.00	0.12	0.04	0.00	0.00	0.00	0.00	0.04	0.03	0.02	0.00	0.00	0.48	0.00	0.00	0.03	0.00
B15	0.00	0.00	0.00	0.02	0.02	0.00	0.05	0.00	0.00	0.09	0.03	0.07	0.12	0.04	0.00	0.23	0.00	0.07	0.00	0.00	0.00	0.14	0.07	0.09
B16	0.00	0.00	0.00	0.00	0.17	0.00	0.00	0.00	0.03	0.12	0.12	0.07	0.06	0.03	0.23	0.00	-0.05	0.06	0.00	0.00	0.03	0.06	0.00	0.00
B17	0.04	0.00	0.03	0.00	0.00	0.00	0.00	0.00	0.03	-0.07	0.00	0.00	-0.06	0.02	0.00	-0.05	0.00	0.03	0.26	0.00	0.08	0.00	0.00	0.00
B18	0.10	0.00	0.00	0.00	0.00	0.00	0.00	0.00	0.02	0.00	0.01	0.00	0.04	0.00	0.07	0.06	0.03	0.00	0.02	-0.02	0.36	0.00	0.00	0.00
B19	0.10	0.00	0.08	0.27	0.00	0.01	0.00	0.00	0.03	0.00	0.00	0.00	0.00	0.00	0.00	0.00	0.26	0.02	0.00	0.02	0.00	0.00	0.00	0.04
B20	0.00	0.22	0.00	0.07	0.00	0.00	0.00	0.00	0.02	0.02	0.00	0.02	0.00	0.48	0.00	0.00	0.00	-0.02	0.02	0.00	-0.04	0.13	0.08	0.00
B21	0.00	0.00	0.00	0.00	0.00	0.00	0.00	-0.02	0.05	0.00	0.17	0.00	0.00	0.00	0.00	0.03	0.08	0.36	0.00	-0.04	0.00	0.00	0.00	0.21
B22	0.00	0.13	0.00	0.00	0.05	0.02	0.00	0.00	0.06	0.13	0.00	0.10	0.03	0.00	0.14	0.06	0.00	0.00	0.00	0.13	0.00	0.00	0.36	0.11
B23	0.00	0.00	0.00	0.00	0.00	0.07	0.06	0.04	0.03	0.03	0.00	0.00	0.05	0.03	0.07	0.00	0.00	0.00	0.00	0.08	0.00	0.36	0.00	0.14
B24	0.02	0.00	0.00	0.00	-0.01	0.00	0.00	0.00	0.00	0.01	0.05	0.00	0.09	0.00	0.09	0.00	0.00	0.00	0.04	0.00	0.21	0.11	0.14	0.00

#### 3.2.4 Network accuracy and stability

**Figure 4:**
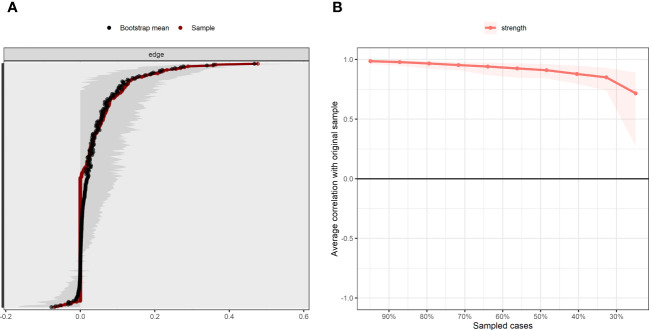
Accuracy and stability of network structure model. **(A)** Accuracy analysis of edge weights. **(B)** Stability analysis of centrality indicators.

**Figure 4A** shows the bootstrapping analysis results for the edge weights. The 95% confidence interval for the bootstrap edge weights is relatively narrow, indicating that the network has strong accuracy. **Figure 4B** illustrates the results of the case-dropping subset bootstrap test. Here, the centrality stability coefficient (CS-C) is 0.673, which is above 0.5. Thus, 67.3% of the samples can be discarded without significant impact on the network structure, indicating that the network structure has strong stability.

## 4 Discussion

Demoralization is an important psychological problem in female cancer patients. Thus, it is critical to understand the complex interactions between specific symptoms in female cancer patients with demoralization to ensure accurate intervention and treatment of their psychological symptoms. To the best of our knowledge, this is the first study based on network analysis to explore the network structure relationships between specific symptoms of demoralization in Chinese female cancer patients. Through network analysis, this study identified core symptoms and bridge symptoms in the network, while focusing on symptoms in the network with the strongest edge connections.

The results showed that “Discouragement (C3)” is the most central symptom of demoralization among female cancer patients, which is manifested by the patient’s internalized feelings of disheartenment. The long cycle of cancer treatment, the need to face a variety of adverse reactions during chemotherapy or radiotherapy, uncertain treatment effects, and the fear of disease development place significant psychological pressure on patients, which can easily lead to depression and discouragement. In addition, female personality traits make such individuals more sensitive to negative events. Studies show that women’s emotional reactions are generally stronger than those of males, making them more prone to serious psychological burdens in the face of diseases (29, 30). This study confirms that “Irritable (B2)” is the most severe symptom of demoralization, “Discouragement (C3)” is the most central symptom in the network. Moreover, we were surprised to find that “Irritable (B2)” is not only the most severe symptom, but also the most prevalent symptom. And the ranking of the top three symptoms in severity is exactly the same as the top three symptoms in prevalence. The results showed inconsistency between the most severe symptoms, most prevalent symptoms and the most central symptoms, presumably due to the fact that “Irritable (B2)”, although the most prevalent and severe problem in women with cancer, has little effect on other symptoms of demoralization. “Discouragement (C3)”, on the other hand, although not the most prevalent symptom, had the strongest influence on other symptoms, suggesting that healthcare professionals should focus on the severity of “Discouragement(C3)” when assessing the mental health status of female cancer patients. The study also found that the symptom most closely connected with “Discouragement (C3)” is “Loneliness (C4)”, which means that “Loneliness (C4)” often appears together with “Discouragement (C3)”and is common in female cancer patients, with both playing a synergistic role in demoralization. The results show that both “Discouragement (C3)” and “Loneliness (C4)” are among the most predictable symptoms. According to the definition of predictability, effective interventions targeting “Loneliness (C4)” can also weaken the degree of “Discouragement (C3)”. A synergistic intervention targeting both symptoms may further improve demoralization. Medical staff should focus on the dual health education of patients and their families, encouraging family members to provide support and companion to patients, which can alleviate patients’ loneliness and frustration.

The second strongest symptom was “No self-worth (A3)”, which indicates a sense of meaninglessness in the face of the illness. Women often take on heavy family-oriented responsibilities such as housework and raising children, but the burden of disease can make them lose this role. Some patients can no longer continue to share the burden with their families, and their physical condition makes them more dependent on their family members for care, increasing the family’s burden (31). In society, most female patients cannot continue to engage in social labor to obtain remuneration for physical reasons. The survey results also show that the proportion of unemployed female cancer patients is significantly higher than that of employed and retired patients. Not being able to create economic benefits for one’s family but having to pay high medical expenses for a long period of time, alongside increased financial and energy burdens on the family can make a patient feel frustrated with her current situation. Due to this frustration, the patient can feel guilty about her family and believe that she no longer has living value (8). It is suggested that healthcare professionals encourage patients to undertake appropriate family and social responsibilities and actively participate in healthcare decision-making. Patients should also establish reasonable goals, formulate life plans, and reflect upon their self-worth through goal realization.

The third strongest symptom was “Don’t want to live (A5)”, which highlights the patient’s painful feelings of despair in the face of cancer and indicates a loss of self- efficacy and will to live. According to the survey, the incidence of suicidal ideation in female cancer patients is slightly higher than that among males (32), which may be influenced by the traditional concept of male superiority over females in China (33). Women in China occupy a vulnerable position, have weak recourse, and lack the awareness to utilize social resources, making them able to seek relief only through suicide (32). Secondly, the cycle of cancer diagnosis, treatment and rehabilitation is long, and adverse reactions increase with prolonged treatment time, making it impossible for patients to perceive a hope of recovery. For patients, these large physical and psychological burdens and a sense of guilt for one’s family can lead to a complete loss of the will to survive. “Don’t want to live (A5)” is also one of the most predictable symptoms. The most closely connected symptom is “No meaning of survival (A4)”, suggesting that medical staff’s intervention in “No meaning of survival (A4)” can reduce the degree of “Don’t want to live (A5)” and thus improve the patient’s will to live (34).

In addition to the core symptoms, network analysis can also infer how the dimensions of Demoralization interact with each other. The results showed that “Hopeless (D4)” had the highest bridge strength, followed by “Discouragement (C3)”, “No self-worth (A3)”, “Poor spirits (E2)”, and “Vulnerability (B3)”, which were identified as bridge symptoms in all dimensions. The edge weights showed that the most closely connected dimensions for demoralization were “Loss of meaning” and “Sense of failure”, which were connected by the bridge symptom “No self-worth” and the symptom “Worthless” (A3—E6, edge weight=0.27). This result suggests that losing a sense of meaning and purpose in life is likely to lead to a strong sense of failure for patients and as a result of self-denial. Deckx et al. (35) found that most cancer survivors experience a moderate level of loneliness, which makes them more concerned about themselves, overly concerned about the meaning of life’s existence, and prone to feelings of uselessness when they perceive that they are not wanted. Secondly, the vast majority of the subjects in this study were female breast cancer patients, and most of the participants were in the stage of chemoradiotherapy. Patients experience symptoms such as hair loss and edema, which can lead to serious disturbances in one’s own image. Patients can also lose important organs and face reduced femininity due to surgery, causing them to have a strong sense of inferiority and failure (36), and believe that life has lost its meaning. Healthcare professionals should especially pay attention to the postoperative psychological state of patients (37) and encourage family members to actively communicate with patients and enhance each patient’s sense of meaning in life by establishing spiritual connections. Such professionals could instruct patients to wear wigs and prosthetic breasts and engage in other forms of self-image management to improve their sense of self-worth. This measure could avoid transitioning from the bridge symptom “No self-worth (A3)” to the symptom “Worthless (E6)”, which may lead to a sense of failure.

The bridge symptom “Poor spirits” and the symptom “Loss of emotional control” (E2-D1, edge weight = 0.22) ranked second in terms of closeness of connection; these symptoms belong to the “Sense of failure” and “Helplessness” dimensions, respectively. Previous research (38–40) found a strong association between physical symptoms such as fatigue and a fear of cancer recurrence among cancer survivors, as patients may misinterpret their fatigue as a sign of cancer recurrence or progression. However, fighting the disease commonly leads to fatigue, causing the patient to fear cancer recurrence and resulting in a poor mental state. This factor also confirms the results of this study. This phenomenon suggests that when a patient’s mental state is obviously poor, it may be a critical period for therapeutic intervention, which can be achieved through physical therapy to alleviate the patient’s fatigue, analgesic therapy to improve the patient’s sleep quality, and dynamic health education to eliminate the patient’s fear of relapse, so as to prevent the patient from being unable to cope with the disease, leading to the occurrence of “Loss of emotional control (D1)”.

The third most closely connected symptoms were “Discouragement” and “Vulnerability” (C3-B3, edge weight = 0.14), which were bridge symptoms of each other, belonging to the dimensions of “Disheartenment” and “Dysphoria”, respectively. This result shows that the two dimensions influence each other through a mutual cause-and-effect relationship. The “Discouragement (C3)” symptom highlights the patient’s negative attitude towards the cancer diagnosis and further triggers negative coping behaviors, leading the patient to acquire a “Vulnerability (B3)” state of mind, which corresponds to a lower level of mental resilience. As Qian’s research (41) shows, “positive attitudes towards reality and the future” and “taking positive actions” play a significant role in improving patients’ resilience. Studies have found that middle-aged women with cancer have lower levels of resilience than men (41), which makes this population more psychologically vulnerable when faced with cancer. If the corresponding emotions are not attended to, they can lead to resentment, as the patient may feel guilty about her family while at the same time believing that she is not valued. This ambivalence of both guilt and dissatisfaction can cause patients to become more sensitive, vulnerable, and subject to bursts of intense negative emotions. In the process of treatment, medical staff should pay special attention to the identification and treatment of “Discouragement (C3)” and “Vulnerability (B3)” to avoid the interactions between bridge symptoms that can produce more serious psychological symptoms. Professional medical measures such as individualized cognitive behavioral therapy can be taken to improve the patient’s mental resilience level and prevent the generation of negative emotions.

The fourth most closely connected dimensions were “Helplessness” and “Loss of meaning”, which were connected by the bridge symptom “Hopeless” and the symptom “No meaning of survival” (D4-A4, edge weight = 0.12). If the patient’s long-term helplessness is not sufficiently resolved, it will eventually lead to a loss of purpose and meaning of life accompanied by a loss of hope for survival (18). The study also found that the symptom “Hopeless (D4)” is not only the fourth strongest symptom in the network but also a bridge symptom, which means that the intervention of the “Hopeless (D4)” symptom can not only weaken most of the connections in the network but also avoid the development of the “Hopeless (D4)” emotion to “No meaning of survival (A4)”. This factor also requires the attention of healthcare professionals.

Regarding the interactions between bridge symptoms in the network structure model, the results also show that the dimensions of “Loss of meaning and Sense of failure”, “Sense of failure and Helplessness”, and “Helplessness and Loss of meaning” are mutually connected by bridge symptoms. Together, these three strongest bridge symptoms form a “Loss of meaning–Sense of failure–Helplessness–Loss of meaning” cycle, indicating that negative demoralization-based emotions will gradually increase and produce stronger negative emotions, eventually leading to a loss of the patient’s willingness to live. This result suggests that healthcare professionals can view “Loss of meaning–Sense of failure–Helplessness–Loss of meaning” as a whole when intervening in female cancer patients with demoralization. Through family narrative therapy, mutual support groups, and other interventions, patients can be encouraged to share their experiences in successfully overcoming the disease. This type of sharing can provide help to others by increasing one’s sense of meaning in survival and reducing feelings of failure, thus breaking the undesirable cycle of “Loss of meaning-Sense of failure–Helplessness–Loss of meaning”.

## 5 Limitations

First, this study adopted a self-reporting method, which may have introduced a self-reporting bias. Secondly, because this was a cross-sectional study, the interpretations of causal relationships are limited. Furthermore, despite some studies showing more optimistic results, there are still a number of studies that clearly show that cross-sectional networks are not necessarily decisive symptom predictors of changes in overall treatment symptoms, even in large datasets (42). Although centrality indices may seem intuitive, easily applicable, and frequently used, the possible problems with interpreting these indices in the context of mental networks have not been examined (43). In future work, longitudinal studies could be used to explore the changes in various dimensions of demoralization among cancer patients over a longer diagnosis period. In addition, due to time constraints, the participants in this study were all from eastern China, with no samples from central and western regions. In the future, multi-center studies with larger samples could be carried out to improve the generalizability of the results and thus more accurately explore the interactive relationships between the symptoms of demoralization among female cancer patients.

## 6 Conclusions

In this study, we analyzed the interactions between symptoms of demoralization using a network structure model and found that the demoralization symptoms of “Discouragement (C3)”, “No self-worth (A3)”, “Hopeless (D4)”, and “Vulnerability (B3)” were both the most central symptoms and those with the highest bridge strength. We suggest that these symptoms represent the core symptoms leading to demoralization and that these core symptoms can influence each other. In future clinical work, the above-mentioned core symptoms and bridge symptoms should be identified, and precise interventions should be carried out to counteract the strongest interactions between symptoms to improve the psychological care effects for female cancer patients.

## Data availability statement

The original contributions presented in the study are included in the article/supplementary materials, further inquiries can be directed to the corresponding author/s.

## Ethics statement

The studies involving humans were approved by Medical Ethics Committee of Jiangsu University. The studies were conducted in accordance with the local legislation and institutional requirements. The participants provided their written informed consent to participate in this study.

## Author contributions

YG: Data curation, Investigation, Writing – original draft, Writing – review & editing. BS: Data curation, Writing – review & editing. JT: Supervision, Writing – review & editing. CL: Supervision, Writing – review & editing. ZB: Supervision, Writing – review & editing. XW: Data curation, Writing – review & editing. TF: Data curation, Writing – review & editing. QZ: Data curation, Writing – review & editing. LL: Data curation, Writing – review & editing. WS: Data curation, Writing – review & editing.
